# Pyogenic Ventriculitis Caused by Listeria Monocytogenes

**DOI:** 10.5334/jbsr.2033

**Published:** 2020-03-06

**Authors:** Fabrice Le Fevere De Ten Hove, Thomas Reumont

**Affiliations:** 1 Université Catholique de Louvain, BE; 2 Hôpital de Jolimont, BE

**Keywords:** Pyogenic ventriculitis, Ventricular empyema, Meningitis, Diffusion restriction, Listeria monocytogenes

## Abstract

In case of meningitis, intraventricular sediments showing diffusion restriction on brain magnetic resonance imaging are highly suggestive of pyogenic ventriculitis for which early diagnosis is crucial for appropriate treatment.

## Case Report

A 78-year-old immunocompetent man presented to the emergency room with high fever and acute confusional state. Neurological examination was normal. Blood analysis revealed markedly increased white blood cell count (19.6 cells/nL [4.0–11.0]) and C-reactive protein (223 mg/L [0.0–10.0]). Computed tomography (CT) of the brain at the time of admission was normal, except for a large left frontal sequela.

A few hours later, his condition deteriorated, with onset of generalized seizures and loss of consciousness. Meningitis was highly suspected and therefore, lumbar puncture was performed. Cerebrospinal fluid (CSF) analysis showed pleocytosis (2080 cells/mm^3^, 88% of polymorphs) and increased protein levels (4.36 g/L [0.1–0.45]), which confirmed the diagnosis of bacterial meningitis. Listeria monocytogenes was cultured from the CSF and blood samples, resulting in intravenous Amoxicillin treatment.

**Figure 1:**
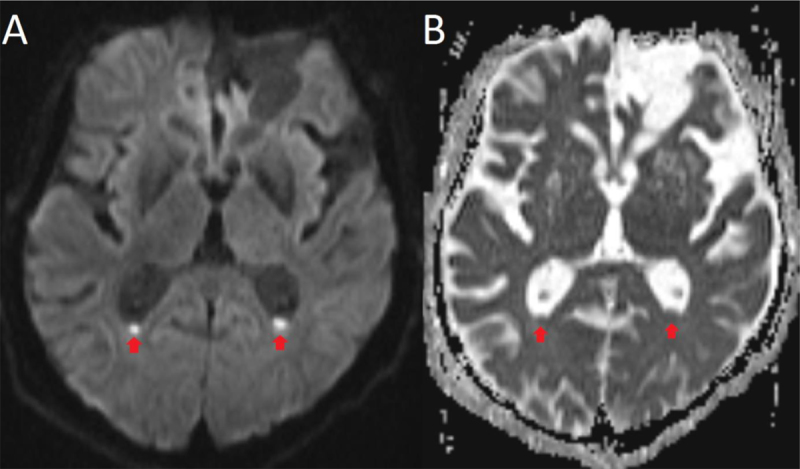


Due to the lack of clinical improvement, brain magnetic resonance imaging (MRI) was performed and revealed hyperintense debris in the occipital horns on diffusion-weighted images (DWI, Figure 1A) with reduced apparent diffusion coefficient (ADC, Figure 1B).

**Figure 2:**
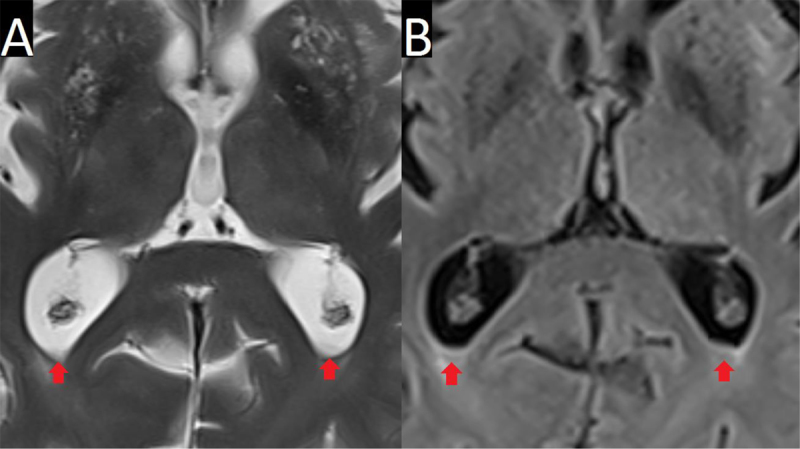


These ventricular sediments were slightly hypointense on T2-weighted images (T2WI, Figure 2A) and on fluid-attenuated inversion recovery (FLAIR, Figure 2B).

**Figure 3:**
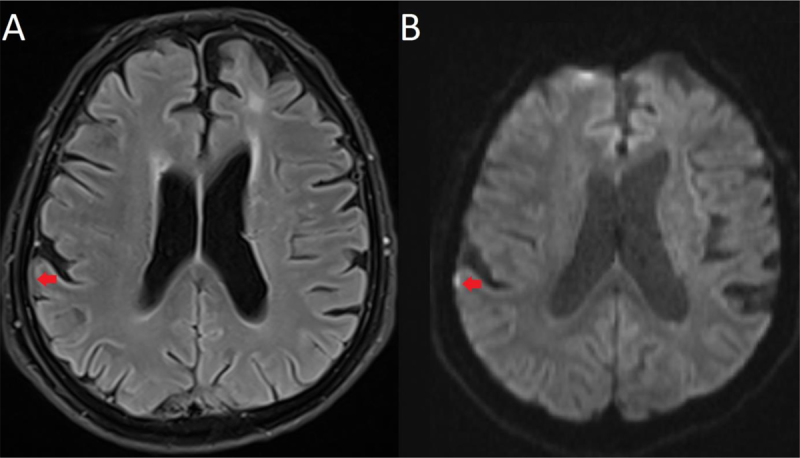


MRI showed a bright spot in the subarachnoid space of the right parietal region on DWI (Figure 3B) appearing slightly hypointense on FLAIR (Figure 3A).

There was no ependymal enhancement nor meningeal thickening on gadolinium enhanced T1-weighted images.

Final diagnosis was pyogenic ventriculitis (PV) and meningitis.

Antimicrobial therapy was adjusted with combination of Amoxicillin and Co-trimoxazol. Treatment lasted eight weeks with good clinical evolution.

## Comment

PV is a rare intracranial infection that mainly affects children and immunocompromised patients. It is characterized by existence of suppurative fluid in the ventricles.

PV has also been described as ventricular empyema, pyocephalus or pyogenic ependymitis.

Primary PV, although extremely rare, occurs after direct hematogenous spread of bacteria to the choroid. PV is more often secondary to extension of meningitis, rupture of brain abscess, penetrating head injury or neurosurgery [1]. If not diagnosed and treated in time, it can lead to severe neurological sequelae or even death. Signs and symptoms are those of meningitis. Neuroimaging techniques are mandatory for the diagnosis, especially MRI, on which intraventricular sediments can be seen. High viscosity of the purulent deposits decreases water molecules mobility, which reduces ADC on DWI. Therefore intraventricular sediments showing diffusion restriction are highly suggestive of PV [1].

Hydrocephalus, ependymal enhancement, periventricular and meningeal abnormalities are other findings that may be helpful, but in our case, only ventricular and meningeal purulent sediments were identified. CSF analysis with isolation of the pathogenic agent is essential in determining the antimicrobial therapy.
